# 4-Dimensional Intracardiac Echocardiography-Guided Antegrade TAVR Using a Novel CABLE-TAVR Technique

**DOI:** 10.1016/j.jaccas.2026.108156

**Published:** 2026-05-13

**Authors:** Kaleb J. Robin, Joshua M. Campbell, Jorge A. Castellanos

**Affiliations:** a Department of Internal Medicine, Louisiana State University Health Sciences Center New Orleans, LSU Internal Medicine Residency, Baton Rouge, Louisiana, USA; b Structural Heart Program, Our Lady of the Lake Heart and Vascular Institute, Baton Rouge, Louisiana, USA

**Keywords:** aortic valve, biotechnology, valve replacement

## Abstract

**Background:**

An antegrade approach to transcatheter aortic valve replacement (TAVR) has become less common because of the complexity and chance of complication, but use of a large-bore sheath to protect the mitral valve has been described.

**Case Summary:**

We present a 75-year-old patient with severe symptomatic aortic stenosis who underwent antegrade TAVR with 4-dimensional intracardiac echocardiography under conscious sedation. We describe a novel technique using an Edwards Commander Delivery System to flex a large-bore sheath across the mitral valve to protect the atrial septum and mitral valve during valve delivery.

**Discussion:**

Patients with limited vascular access for retrograde TAVR and who are not candidates for general anesthesia owing to pulmonary disease can undergo antegrade TAVR under conscious sedation and 4-dimensional intracardiac echocardiography guidance.

**Take-Home Message:**

Our case illustrates an antegrade approach with a novel technique that may be a suitable alternative to TAVR in high-risk patients who otherwise would not be candidates for aortic valve replacement.

## Visual Summary

**Figure:**
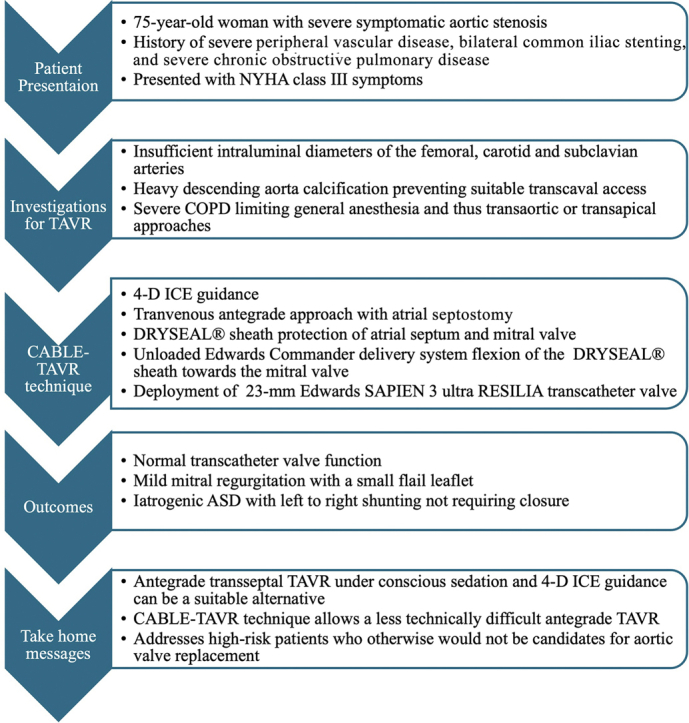

**Visual Summary:**
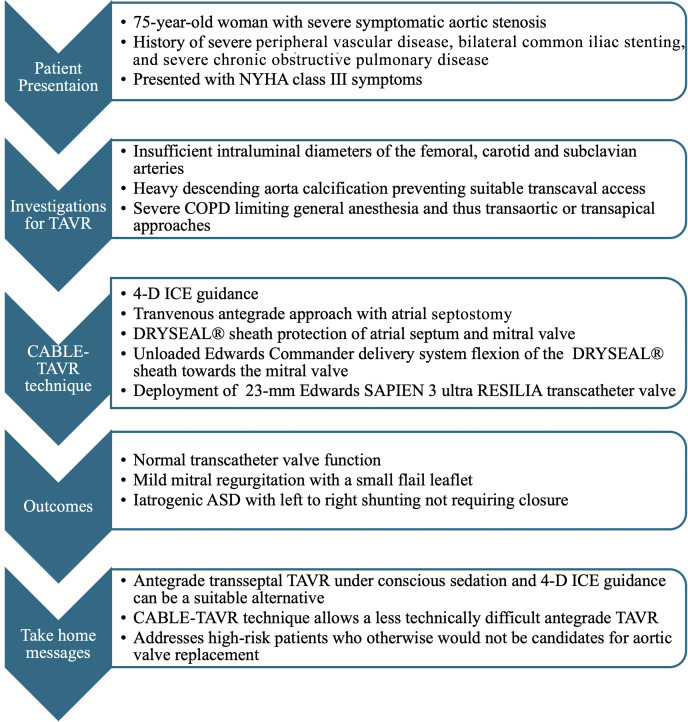
Case Overview for the Novel CABLE-TAVR Technique

## History of Present Illness

A 75-year-old woman presented for NYHA class III symptoms. Echocardiography revealed severe aortic stenosis (maximum velocity of 5.0 m/s, mean pressure gradient of 56 mm Hg, peak pressure gradient of 100 mm Hg, and aortic valve area of 0.53 cm^2^).

**Take-Home Messages**

• The antegrade trans-septal approach to TAVR with the patient under conscious sedation and 4-D ICE guidance can be a suitable alternative approach for high-risk patients who otherwise would not be candidates for aortic valve replacement.
• Correct material selection and the novel CABLE-TAVR technique allow a less technically difficult antegrade TAVR.

## Past Medical History

She had severe symptomatic aortic stenosis, severe peripheral vascular disease with past bilateral carotid endarterectomy and bilateral common iliac stenting, severe chronic obstructive pulmonary disease on supplemental oxygen, paroxysmal atrial fibrillation, hypertension, history of tobacco use, and dyslipidemia. She had undergone aortic valvuloplasty for severe symptomatic aortic stenosis and experienced improved functional status for 1 year before symptom recurrence.

## Investigations

**Figure 1:**
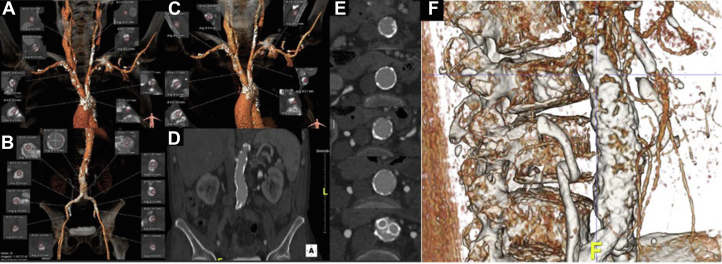
Preoperative Vascular Imaging Preoperative computed tomography angiography with 3-dimensional reconstruction of the (A) common carotid artery, (B) iliofemoral artery, and (C) subclavian artery exhibiting narrow internal diameters. Heavy continuous calcification of the descending aorta is shown on coronal (D) and axial (E) preoperative computed tomography angiography imaging. The lateral view of the descending aorta on 3-dimensional reconstruction shows lack of an adequate calcium-free window for transcaval access (F).

Transcatheter valvular replacement options from a retrograde approach were explored with computed tomography angiography (Figure 1). Imaging revealed previous bilateral common iliac stenting and insufficient intraluminal diameters of the femoral, carotid, and subclavian arteries for device delivery system transversal. Lack of an adequate calcium-free window of the descending aorta also prevented suitable transcaval access. Because of her severe comorbidities, including severe obstructive pulmonary disease (ratio of forced expiratory volume in 1 second to forced vital capacity: 54%; forced expiratory volume in 1 second: 46% predicted), she was not deemed to be a candidate for general anesthesia by the heart team and thus not a candidate for transaortic or transapical approaches. The heart team determined there was a reasonable chance of improved quality of life with valve replacement, so the team elected for an antegrade transvenous approach.

## Management

**Figure 2:**
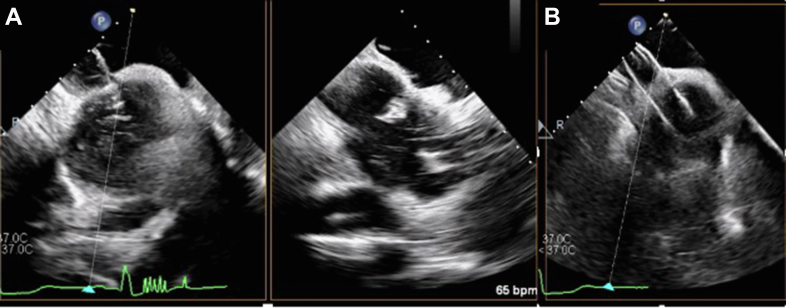
Trans-septal Puncture Intracardiac echocardiography showing the trans-septal puncture (A) followed by atrial septostomy (B).

**Figure 3:**
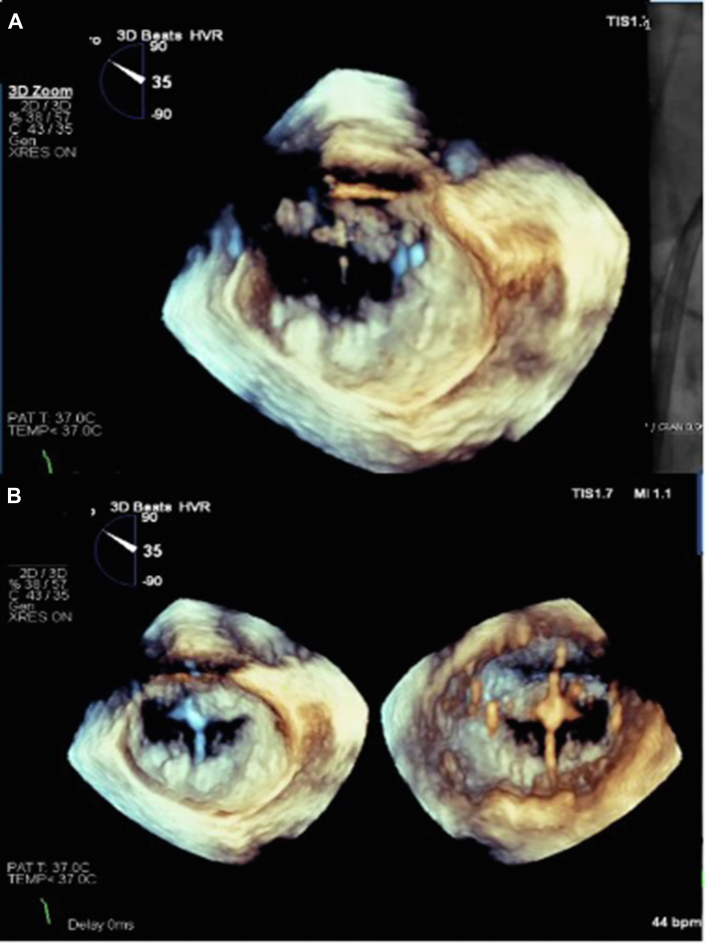
Mitral Valve Intracardiac Echocardiography Four-dimensional intracardiac echocardiographic view of the mitral valve from the left atrium (A) before and (B) after wire placement.

**Figure 4:**
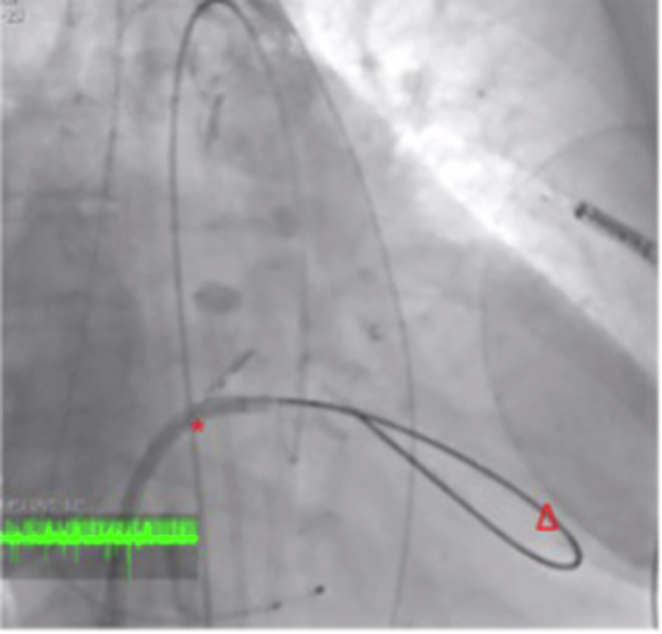
Aortovenous Loop Creation Fluoroscopic image with the Agilis introducer (∗) positioned toward the mitral valve with a balloon-tipped catheter (Δ), creating a loop within the left ventricle and out of the aortic outflow tract.

Given the patient's poor respiratory reserve and intubation risk, the team placed the patient under conscious sedation with monitored anesthesia care and 4-dimensional (4-D) intracardiac echocardiography (ICE) guidance. Bilateral femoral venous access was first obtained. A 16-F, 35-cm sheath (Cook Medical) was placed in the right femoral vein. A 10-F, 25-cm PINNACLE sheath (Terumo Medical Corporation) was placed in the left femoral vein for advancement of a VeriSight Pro ICE catheter (Philips) into the right atrium. Bilateral femoral arterial access was then obtained. A 5-F Terumo PINNACLE sheath was advanced through the right femoral artery for insertion of a 5-F pigtail catheter (Boston Scientific) for root angiography. A 6-F, 45-cm Terumo PINNACLE DESTINATION guiding sheath was placed into the left femoral artery for placement of a snare catheter. Full-dose heparin was then administered for anticoagulation. Using 4-D ICE, a trans-septal puncture was performed by a Brockenbrough needle using an SL-1 sheath (Abbott) in the usual manner. An atrial septostomy was performed using a 14 × 40-mm Armada 35 peripheral balloon (Abbott) (Figure 2). After the balloon was removed, the ICE catheter was advanced across the septum to image the mitral valve from the left atrium (Figure 3). The SL-1 sheath was exchanged for a steerable Agilis introducer (Abbott) that was advanced through the septostomy and positioned toward the mitral valve. A balloon-tipped 6-F catheter was advanced through the Agilis introducer, mitral valve, left ventricle, and ultimately through the aortic valvular apparatus (Figure 4). For aortovenous loop creation, a flexible-shaft 0.035-in × 400-cm Nitrex guidewire (N354002, Medtronic) was advanced through the balloon-tipped catheter and into the aorta with ultimate externalization from the left femoral artery after snaring by an Amplatz gooseneck snare (Medtronic).

**Figure 5:**
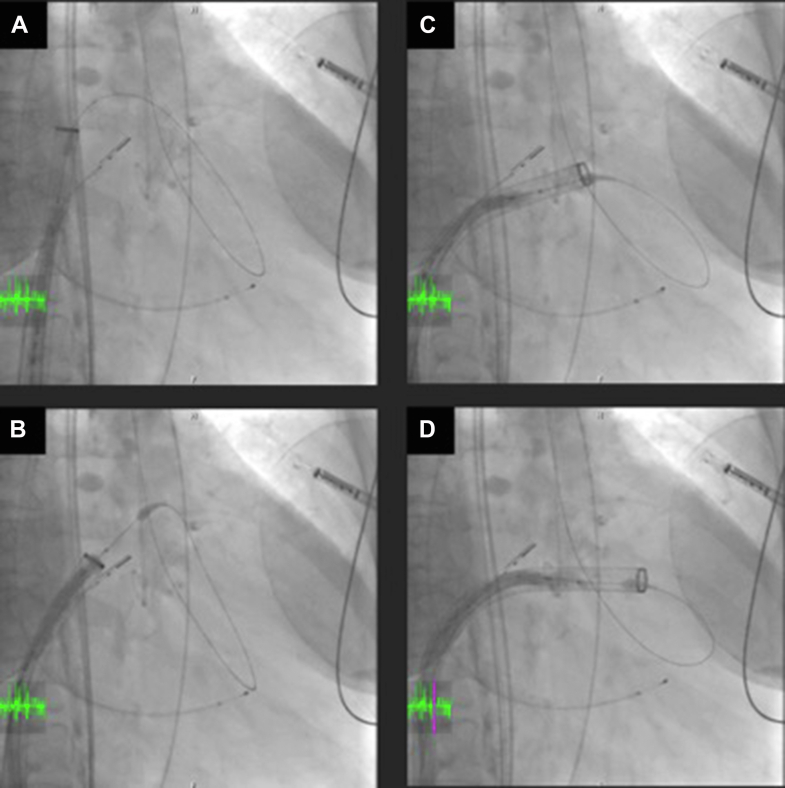
CABLE-TAVR Technique Fluoroscopic images of the CABLE-TAVR technique in sequence (A to D) showing an unloaded Edwards Commander delivery system bending the overlying DRYSEAL sheath toward the mitral valve.

**Figure 6:**
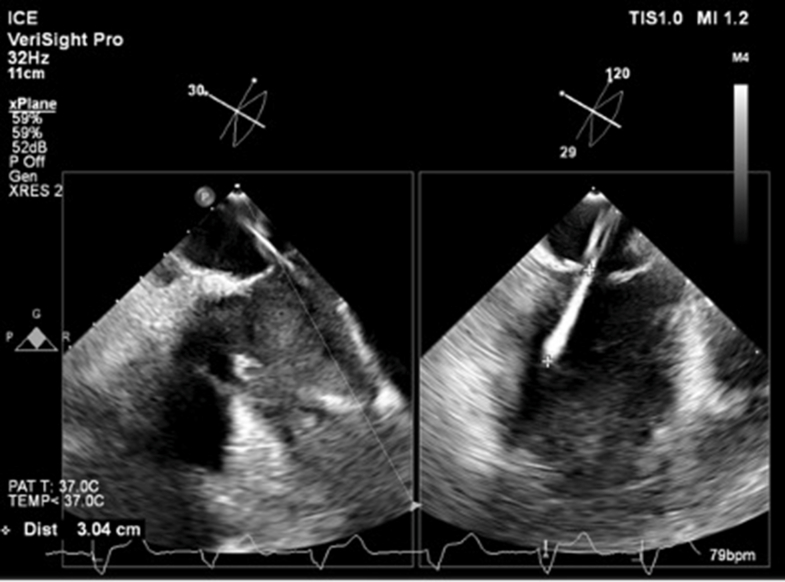
DRYSEAL Sheath Across the Mitral Valve Intracardiac echocardiography showing the DRYSEAL sheath traversing through the mitral valve with its distal tip in the midcavity of the left ventricle.

**Figure 7:**
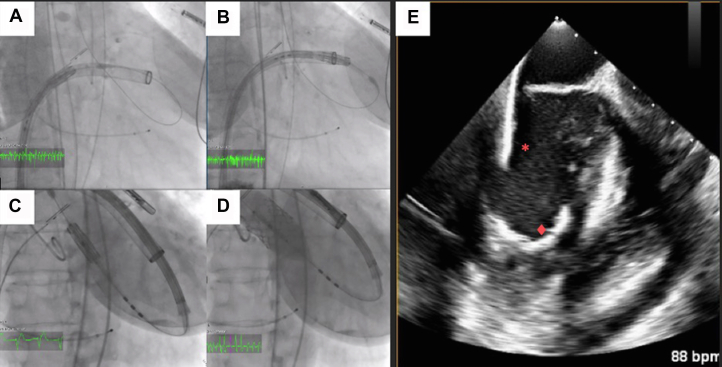
Prosthetic Aortic Valve Delivery Fluoroscopic images of the Edwards Commander delivery system loaded with the prosthetic valve within the DRYSEAL sheath (A), at the distal end of the sheath (B), within the aortic valve apparatus (C), and at deployment (D). Intracardiac echocardiographic images show the DRYSEAL sheath in the left ventricular midcavity (∗) with the prosthetic valve (♦) at the apex curving superiorly toward the aortic outflow tract (E).

The balloon-tipped catheter and Agilis introducer were removed, and the 16-F sheath in the right femoral vein was exchanged for a 26-F, 65-cm DRYSEAL sheath (WL Gore & Associates). The sheath was then advanced through the atrial septum but tended to track to the roof of the left atrium rather than toward the mitral valve. An unloaded Edwards Commander delivery system was advanced within and toward the distal end of the DRYSEAL sheath. Using the novel CABLE-TAVR (Commander-Assisted Bending of Large-borE sheath for antegrade transcatheter aortic valve replacement [TAVR]) technique, flexion of the Commander device within the sheath allowed angling of the sheath toward the mitral valve apparatus (Figure 5, Video 1). The sheath was advanced across the mitral valve under ICE guidance to provide a pathway for the valve delivery system (Figure 6). After placement of the sheath tip within the midcavity of the left ventricle, the Commander device was removed. A 23-mm Edwards SAPIEN 3 ultra RESILIA transcatheter valve was crimped outside of the body and loaded onto the delivery system. The delivery system was inserted through the sheath to its tip, through the left ventricle, and into the aortic valve apparatus (Figure 7). A 6-F Boston Scientific multipurpose catheter was inserted through the left femoral artery access and used to interact with the nose cone of the Commander device to prevent aortic movement during valve deployment.

## Outcome and Follow-Up

**Figure 8:**
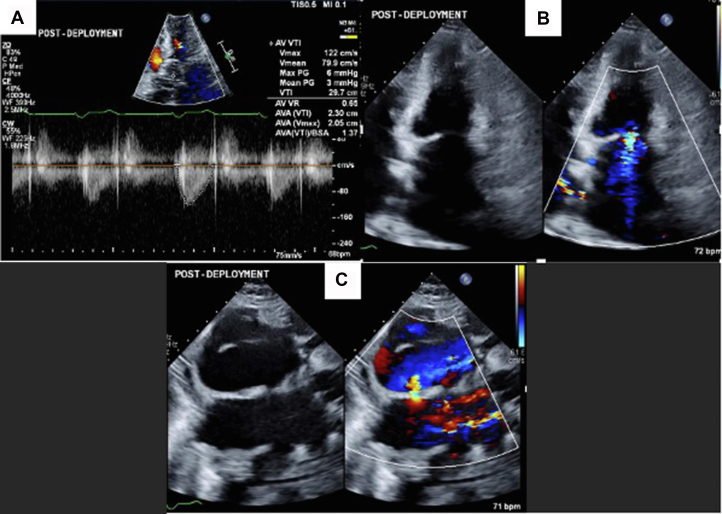
Postoperative Echocardiography Postoperative transthoracic echocardiography showing normal function of the prosthetic aortic valve (A), mild mitral regurgitation (B), and a small atrial septal defect with left to right shunting (C).

The valve was successfully deployed. Wires and sheaths were removed with no indication of major intraoperative complications. Postdeployment valvular assessment indicated normal transcatheter valve function. Mild mitral regurgitation with a small flail leaflet and an iatrogenic atrial septal defect with left to right shunting not requiring closure were noted postoperatively (Figure 8). The patient had no hospital readmission for decompensated heart disease for 1 year after the intervention.

## Discussion

Cribier et al^1^ profoundly changed the landscape of interventional cardiology in 2002 when they performed the first TAVR in Rouen, France. The antegrade trans-septal approach was successful in his very sick patient who had a severely calcified bicuspid aortic valve presenting with cardiogenic shock. The antegrade approach to TAVR has become less common because of its complexity and the risk of complications. Despite the heightened preoperative morbidity among the earliest patients enrolled for TAVR, most early patients experienced favorable outcomes.^2^ The retrograde approach to TAVR was developed and first completed in 2005 to address concern for the risks imposed by an antegrade approach, namely, formation of an atrial septal defect, perceived risk of tamponade with use of trans-septal puncture, and potential damage to the mitral valve.^3^ In subsequent years, use of an antegrade approach was largely sidelined to patients without adequate arterial access for retrograde TAVR.^4^ Alternative access techniques including carotid, transapical, and transaortic techniques have been developed but often require general anesthesia. The transcaval approach can be limited by the lack of an inferior vena cava to aortic window because of heavy aortic calcification. Complex vascular disease and clinically significant aortic stenosis often coexist, highlighting the need for more awareness about specific antegrade techniques that have been proven successful. An antegrade trans-septal approach was necessary for this case because of the patient’s inadequate arterial access and inability to tolerate general anesthesia due to severe lung disease.

To address the risk of atrial septal and valvular damage caused by the traversal of the valve delivery device, an antegrade approach was described that used a DRYSEAL Flex sheath across the mitral valve. The technique reported maintenance of hemodynamic stability during the procedure and development of only mild mitral regurgitation postoperatively.^5^ We used a DRYSEAL sheath in our case but encountered difficulty with navigation across the sharp angle created by the direction of the septostomy and mitral valve position. Our technique used an unloaded Commander delivery system to guide the DRYSEAL sheath safely across the mitral valve by allowing flexion of the distal end of the sheath. To our knowledge, this technique has not been previously described and highlights the need for the development of tools dedicated to the antegrade approach. The U.S. Food and Drug Administration recently approved the SAPIEN M3 mitral valve delivery system (Edwards Lifesciences), which uses a flexible 29-F guide sheath that may be beneficial for these cases.

Although early cases from both antegrade and retrograde TAVR approaches used local anesthesia and mild sedation, general anesthesia is now often used to allow for intraoperative transesophageal echocardiography or certain TAVR approaches. A significant subgroup of patients exists with indication for aortic valvular replacement who have contraindication for general anesthesia because of comorbid conditions. It is imperative that the heart team determines if valve replacement will offer improvement in quality of life to avoid futile intervention. In this case, symptomatic improvement with a previous aortic valvuloplasty illustrated that aortic disease was a significant cause of her symptoms. Previous cases have described the successful use of ICE as a substitute for standard transesophageal echocardiography.^6^^,^^7^ The use of intraoperative transesophageal echocardiography has emerged to confirm positioning of surgical devices, assess potential complications, and monitor prosthetic device function after deployment.^8^ In our case, local anesthesia with 4-D ICE for intraoperative guidance provided adequate intraoperative images and avoided potential complications with general anesthesia in our patient with severe lung disease.

## Conclusions

Preoperative vascular and pulmonary comorbidities among many patients with severe symptomatic aortic stenosis in need of TAVR can prohibit surgical access for retrograde TAVR and the use of general anesthesia. Because of the limited experience with antegrade TAVR, it is important to learn correct equipment and antegrade techniques to address this group of severely ill patients who require antegrade TAVR. The novel CABLE-TAVR technique allows easier placement of a large-bore sheath to effectively protect the atrial septum and mitral valve without significant postoperative complications. Finally, expertise in ICE allows careful intraoperative monitoring and can serve as a suitable alternative for transesophageal echocardiography.

## Funding Support and Author Disclosures

Dr Castellanos is a proctor and consultant with Edwards Lifesciences and local principal investigator for the ENCIRCLE trial with Edwards Lifesciences, with Ancora Heart, and for the CATALYST trial (Abbott). All other authors have reported that they have no relationships relevant to the contents of this paper to disclose.

## Appendix

Video 1 CABLE-TAVR Technique Flexion of the unloaded Commander device is shown within the DRYSEAL sheath, allowing manipulation of the sheath toward the mitral valve.
